# Prediction model construction for the occurrence of LARS after neoadjuvant therapy combined with laparoscopic total mesorectal excision in male patients with mid-low rectal cancer

**DOI:** 10.3389/fonc.2024.1492245

**Published:** 2024-12-13

**Authors:** Deng-Chao Wang, Xue-Feng Peng, Miao Yu

**Affiliations:** ^1^ Department of General Surgery, Zigong Fourth People’s Hospital, Zigong, Sichuan, China; ^2^ Department of Basic Medicine, Sichuan Vocational College of Health and Rehabilitation, Zigong, Sichuan, China

**Keywords:** male, neoadjuvant therapy, rectal cancer, LARS, prediction model

## Abstract

**Background:**

Neoadjuvant chemoradiotherapy for rectal cancer improves surgical outcomes and reduces recurrence but can cause low anterior resection syndrome (LARS), affecting quality of life. This study aims to predict the risk of LARS in male patients with mid-low rectal cancer after laparoscopic total mesorectal excision (TME).

**Methods:**

Clinical data from 203 male patients with mid-low rectal cancer who underwent neoadjuvant therapy and laparoscopic resection were collected. Patients were divided into training (n=143) and validation (n=60) cohorts. LARS risk factors were identified using logistic regression, and a predictive model was constructed and validated using ROC curve, Hosmer-Lemeshow test, calibration curve, and decision curve analysis (DCA).

**Results:**

LARS occurred in 53.6% of the patients in this study. Multivariate logistic regression analysis revealed that BMI ≥ 25 kg/m², tumor distance from the anal margin < 5 cm, radiotherapy, and anastomotic leakage were independent risk factors for postoperative LARS in patients (P < 0.05). The areas under the ROC curves for the training cohort and validation cohort were 0.866 (95% CI: 0.807-0.925) and 0.724 (95% CI: 0.595-0.853), respectively, with both groups showing good goodness-of-fit test results (P > 0.05). The DCA curve indicated that the model had a high clinical utility.

**Conclusions:**

BMI ≥ 25 kg/m², tumor distance from the anal margin < 5 cm, radiotherapy, and anastomotic leakage are independent risk factors for the occurrence of LARS after neoadjuvant therapy combined with laparoscopic TME in male patients with mid-low rectal cancer. These factors should be emphasized in clinical practice, and corresponding preventive measures should be promptly implemented.

## 1 Introduction

Rectal cancer, as a common malignant tumor, has always been the focus of clinical research. In recent years, the widespread application and in-depth study of neoadjuvant chemoradiotherapy in the treatment of locally advanced rectal cancer (LARC) have brought about positive changes in this field, aiming to improve the thoroughness of surgical resection, alleviate postoperative symptoms, and reduce the rate of postoperative recurrence (1, 2). Although there is some controversy over the specific regimen of neoadjuvant chemoradiotherapy, it has become the standard mode of comprehensive treatment for LARC. Neoadjuvant chemoradiotherapy affects the choice of surgical approach, resulting in tumor shrinkage or even downstaging, creating the possibility of sphincter preservation, and combined with laparoscopic surgery, minimizing trauma (3). With the prolongation of overall survival and the increase in sphincter preservation rate among LARC patients, more detailed studies on patient quality of life and rectal-anal function have gradually become the focus of attention. Although sphincter-preserving surgery has a positive psychological impact on patients, a considerable proportion of patients will experience low anterior resection syndrome (LARS) after sphincter-preserving radical surgery, severely affecting their quality of life (4, 5). With the development of surgical techniques and neoadjuvant therapy, we are facing an increasingly complex treatment decision-making environment. While existing studies have identified several common predictive factors for LARS, such as BMI, tumor distance, and radiotherapy, these studies predominantly focus on mixed-gender cohorts, potentially overlooking important sex-specific differences. Male rectal cancer patients, due to unique anatomical features (e.g., narrower pelvis) and a higher risk of fibrosis from radiotherapy, face distinct challenges that may influence LARS outcomes (17, 18, 23). However, research specifically targeting this subgroup remains scarce, limiting clinicians’ ability to make gender-tailored predictions and interventions. In this study, we retrospectively analyzed male patients with mid-low rectal cancer who underwent neoadjuvant therapy combined with laparoscopic radical low anterior resection. By constructing a predictive model based on male-specific risk factors, this study addresses a critical gap in LARS research by focusing on the unique challenges faced by male rectal cancer patients. The model not only identifies independent risk factors but also visualizes these risks through a practical nomogram, providing clinicians with a valuable tool for early risk stratification and personalized management. This approach enables more precise and actionable clinical decision-making, ultimately contributing to improved treatment outcomes and quality of life for patients.

## 2 Methods

### 2.1 Study population

Clinical data of male patients with mid-low rectal cancer who underwent neoadjuvant therapy combined with laparoscopic radical low anterior resection in the Department of General Surgery from January 2017 to December 2022 were retrospectively collected. All patients underwent laparoscopic TME with sphincter preservation and prophylactic stoma formation. Inclusion criteria were as follows: (1) Pathologically confirmed primary rectal cancer, tumor distance from the anal verge is less than 10 centimeters; (2) Age greater than 18 years old; (3) The preoperative integrity and function of the anal sphincter were normal, and there were no symptoms of fecal incontinence. (4) Complete medical records with comprehensive postoperative follow-up data. Exclusion criteria were as follows: (1) Patients with mental disorders; (2) Patients with concomitant malignant tumors such as liver cancer, gastric cancer, etc.; (3) Patients on long-term medication that may affect anal defecation function and intestinal function; (4) Patients undergoing concomitant organ resection during TME; (5) Patients undergoing Hartmann or abdominoperineal resection; (6) Lack of complete follow-up data and clinical pathology information.

### 2.2 Treatment regimen

Neoadjuvant chemotherapy and chemoradiotherapy were administered following standard treatment protocols for patients with locally advanced rectal cancer (stage II and III), such as those with lymph node involvement (N1 or higher) or T3/T4 tumors. Neoadjuvant chemotherapy and chemoradiotherapy regimens: Oxaliplatin is administered intravenously at 130 mg/m² on Day 1; Capecitabine is taken orally at 1000 mg/m² from Day 1 to Day 14, within a 3-week cycle. Patients undergo three such cycles, followed by a two-week interval before proceeding with laparoscopic TME. The chemoradiotherapy regimen includes a total radiation dose of 50 Gy, delivered in daily 2 Gy fractions to the pelvis over 25 sessions, with the chemotherapy component mirroring the neoadjuvant chemotherapy regimen. Laparoscopic TME is then performed 4-8 weeks after completing radiotherapy, integrating both therapeutic modalities in the treatment protocol. Postoperative patients will continue with the same chemotherapy regimen as used preoperatively. Postoperative chemotherapy typically starts 4 weeks after surgery, with timing adjustments based on the patient’s postoperative recovery status and overall condition.

### 2.3 Evaluation of LARS

In this study, a standard LARS questionnaire was administered to patients, consisting of five questions (including gas incontinence, liquid-solid incontinence, fecal frequency, urgency, and 1-hour bowel frequency) (6). Based on the responses to the questionnaire, patients were scored from 0 to 42. According to the obtained LARS scores, patients were divided into three groups: non-LARS group (0-20), mild LARS group (21-29), and severe LARS group (30-42). The LARS questionnaire was first administered at 1 month postoperatively and follow-up assessments were conducted at 3 months, 6 months, and 12 months postoperatively to monitor symptom changes.

### 2.4 Follow-up methods

Follow-up at 12 months postoperatively was conducted using various methods including telephone calls, text messages, WeChat, outpatient visits, or hospitalizations to complete the LARS questionnaire scoring.

### 2.5 Statistical methods

Data statistical analysis was performed using SPSS 23.0. Count data were converted into percentages (%), and chi-square tests were used for analysis. Factors with significant differences were subjected to logistic regression multivariate analysis. Patients included in the study were randomly divided into training and validation cohorts at a ratio of 7:3. The training cohort (also referred to as the experimental group) consisted of 70% of the total study population. This cohort was used to develop the predictive model, where independent risk factors were identified and a nomogram was constructed using multivariate logistic regression analysis. The training cohort was used for model development, and the predictive model was built based on the data from this group. The validation cohort (also referred to as the testing group) consisted of the remaining 30% of the total population. This cohort was used to test and validate the performance of the model developed using the training cohort. Based on the results obtained from the logistic multivariate regression analysis, a nomogram prediction model for predicting the occurrence of LARS was constructed using R software (version R4.2.2). A receiver operating characteristic (ROC) curve was plotted, and the area under the ROC curve (AUC) was calculated to validate the model’s discriminative ability. The calibration of the model was evaluated based on the calibration curve, and the Hosmer-Lemeshow goodness-of-fit test was conducted. Clinical utility of the model was assessed using clinical decision curve analysis (DCA), with a significance level cohort at P < 0.05.

## 3 Results

### 3.1 Incidence of LARS

A total of 203 patients meeting the inclusion criteria were included in this study. Among them, 109 patients developed LARS, with an incidence rate of 53.6%. Among these, 75 cases were classified as mild, accounting for 68.8% of the total incidence, while 34 cases were classified as severe, accounting for 31.2% of the total incidence. During the follow-up period, the incidence rates of both mild and severe LARS showed a decreasing trend over time. General demographic data of the LARS and non-LARS groups are provided in **Table 1**.

**Table 1:** Comparison of general characteristics between LARS and non-LARS groups.

Clinical variables	LARS group (n = 109)	Non-LARS group (n = 94)	χ^2^ value	P value
Age			0.395	0.530
<60 years	57 (52.3)	45 (47.9)		
≥60 years	52 (47.7)	49 (52.1)		
BMI			11.995	< 0.001
<25 kg/m²	74 (67.9)	83 (88.3)		
≥25 kg/m²	35 (32.1)	11 (11.7)		
Diabetes			0.29	0.590
No	86 (78.9)	77 (81.9)		
Yes	23 (21.1)	17 (18.1)		
Radiotherapy			39.905	< 0.001
No	23 (21.1)	61 (64.9)		
Yes	86 (78.9)	33 (35.1)		
Tumor distance from the anal verge			51.577	< 0.001
≥5 cm	24 (22)	68 (72.3)		
<5 cm	85 (78)	26 (27.7)		
Anastomotic leakage			4.508	0.034
No	97 (89)	91 (96.8)		
Yes	12 (11)	3 (3.2)		
Surgery duration			17.804	< 0.001
<3 hours	35 (32.1)	58 (61.7)		
≥3 hours	74 (67.9)	36 (38.3)		
TNM staging			0.105	0.746
Stage II	66 (60.6)	59 (62.8)		
Stage III	43 (39.4)	35 (37.2)		
Tumor differentiation			1.463	0.481
Poorly differentiated	22 (20.2)	13 (13.8)		
Moderately differentiated	46 (42.2)	44 (46.8)		
Well differentiated	41 (37.6)	37 (39.4)		
Lymph node metastasis			0.093	0.760
No	58 (53.2)	48 (51.1)		
Yes	51 (46.8)	46 (48.9)		
Tumor diameter			1.96	0.161
<5 cm	30 (27.5)	18 (19.1)		
≥ 5cm	79 (72.5)	76 (80.9)		
Preservation of the left colonic artery			1.377	0.241
No	78 (71.6)	74 (78.7)		
Yes	31 (28.4)	20 (21.3)		
Time to ileostomy closure			1.231	0.267
≤ 2 months	81 (74.3)	76 (80.9)		
> 2 months	28 (25.7)	18 (19.1)		

### 3.2 Univariate analysis of postoperative LARS

Univariate analysis revealed statistically significant differences between the LARS and non-LARS groups in terms of body mass index, tumor distance from the anal verge, presence of radiotherapy, presence of anastomotic leakage, and surgery duration (P < 0.05), as shown in **Table 2**.

**Table 2:** Univariate analysis of LARS incidence in the training cohort.

Clinical variables	LARS group (n=80)	Non-LARS group (n=63)	χ^2^ value	P value
Age				
<60 years	45 (56.2)	30 (47.6)	1.052	0.305
≥60 years	35 (43.8)	33 (52.4)		
BMI				
<25 kg/m²	54 (67.5)	56 (88.9)	9.083	0.003
≥25 kg/m²	26 (32.5)	7 (11.1)		
Diabetes				
No	63 (78.8)	52 (82.5)	0.3215	0.571
Yes	17 (21.2)	11 (17.5)		
Radiotherapy				
No	15 (18.8)	45 (71.4)	40.161	<0.001
Yes	65 (81.2)	18 (28.6)		
Tumor distance from the anal verge				
≥5 cm	15 (18.8)	48 (76.2)	47.181	<0.001
<5 cm	65 (81.2)	15 (23.8)		
Anastomotic leakage				
No	69 (86.2)	61 (96.8)	4.769	0.029
Yes	11 (13.8)	2 ( 3.2)		
Surgery duration				
<3 hours	28 (35.0)	40 (63.5)	11.472	0.001
≥3 hours	52 (65.0)	23 (36.5)		
TNM staging				
Stage II	50 (62.5)	38 (60.3)	0.070	0.790
Stage III	30 (37.5)	25 (39.7)		
Tumor differentiation				
Poorly differentiated	15 (18.8)	10 (15.9)	1.246	0.2643
Moderately differentiated	37 (46.2)	27 (42.9)		
Well differentiated	28 (35.0)	26 (41.3)		
Lymph node metastasis				
No	44 (55.0)	32 (50.8)	0.250	0.617
Yes	36 (45.0)	31 (49.2)		
Tumor diameter				
<5 cm	21 (26.2)	11 (17.5)	1.567	0.211
≥ 5cm	59 (73.8)	52 (82.5)		
Preservation of the left colonic artery				
No	57 (71.2)	49 (77.8)	0.636	0.376
Yes	23 (28.7)	14 (22.2)		
Time to ileostomy closure			0.459	0.498
≤ 2 months	61 (76.2)	51 (81.0)		
> 2 months	19 (23.8)	12 (19.0)		

### 3.3 Multivariate analysis

The factors with differences identified in the univariate analysis were included as independent variables, with the occurrence of LARS in patients as the dependent variable, to further conduct binary logistic regression analysis. Variables considered in the multivariate analysis included BMI, radiotherapy, tumor distance from the anal verge, anastomotic leakage, and surgery duration. The results indicated that BMI ≥ 25 kg/m², tumor distance from the anal verge < 5cm, radiotherapy, and anastomotic leakage were independent risk factors for the occurrence of LARS in male patients with mid-low rectal cancer undergoing neoadjuvant therapy combined with laparoscopic TME (P < 0.05), as shown in **Table 3**.

**Table 3:** Multifactorial logistic regression analysis of LARS.

Clinical variables	β	SE	Wald χ^2^	OR	95%CI	P value
BMI ≥ 25 kg/m²	1.938	0.672	8.323225	6.947	1.862~25.921	0.004
Radiotherapy	2.126	0.723	8.649481	8.382	2.032~34.573	0.003
Tumor distance from the anal verge <5 cm	1.843	0.537	11.79236	6.313	2.206~18.069	0.001
Anastomotic leakage	2.149	1.028	4.3681	8.573	1.143~64.292	0.037

### 3.4 Establishment and validation of the nomogram prediction model

**Figure 1:**
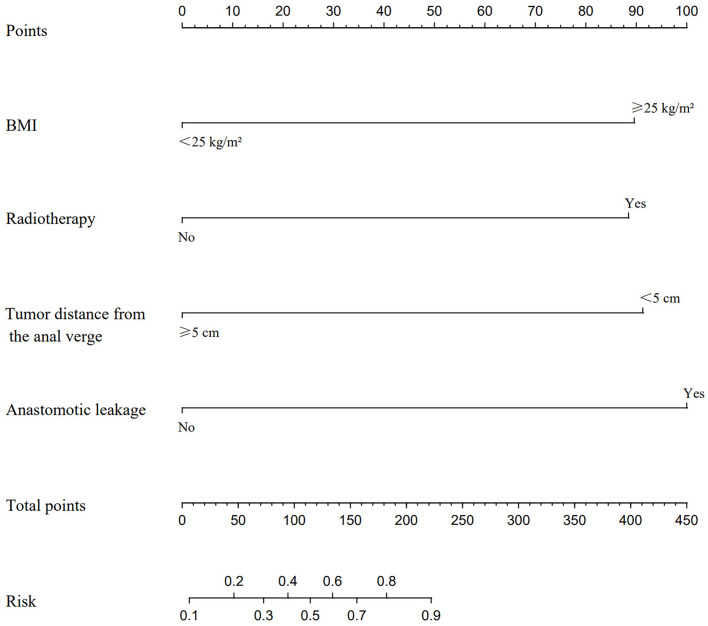
A nomogram model was constructed based on the independent risk factors identified by multivariate logistic regression analysis. This model is utilized to locate the position of each variable on the corresponding axis. Initially, a vertical line is drawn for each variable of the patient to the Points axis to indicate the score of each variable. Subsequently, the scores of all variables read on the Points scale are summed up to obtain Total Points. Finally, a vertical line is drawn from the Total Points axis to determine the risk of LARS at the lower line of the nomogram.

A nomogram prediction model for predicting the occurrence of LARS was constructed using the independent risk factors identified in the logistic multivariate regression analysis. In the nomogram, vertical lines are drawn at the corresponding score positions on the scales for each variable axis. The total score of the variables is then calculated, and the corresponding value at the point on the predicted probability scale corresponds to the risk of developing LARS after sphincter-preserving surgery in male patients with mid-low rectal cancer undergoing neoadjuvant therapy combined with laparoscopic TME. According to the Nomogram prediction model, a score of 89 points is assigned for BMI ≥ 25 kg/m², 88 points for preoperative radiotherapy, 91 points for tumor distance from the anal verge < 5 cm, and 100 points for anastomotic leakage, as shown in **Figure 1**. Case Example: A 65-year-old male patient with a BMI of 28 kg/m², no history of radiotherapy, a tumor located 4 cm from the anal verge, and no postoperative anastomotic leakage. According to the nomogram, we first locate each variable (BMI, radiotherapy history, tumor distance, and anastomotic leakage) on the corresponding axis, draw a vertical line to the Points axis, and record the scores. The score for a BMI of 28 kg/m² (≥25 kg/m²) corresponds to 89 points, the score for no history of radiotherapy corresponds to 0 points, the score for a tumor located 4 cm from the anal verge (<5 cm) corresponds to 91 points, and the score for no anastomotic leakage corresponds to 0 points. Therefore, the total score for this patient is 89 + 0 + 91 + 0 = 180. A vertical line is then drawn from the total score of 180 to the “Risk” axis at the bottom, which gives a risk value of 80%, indicating that the patient has a high risk of developing LARS postoperatively.

### 3.5 Predictive value of LARS occurrence in training and validation cohorts

**Figure 2:**
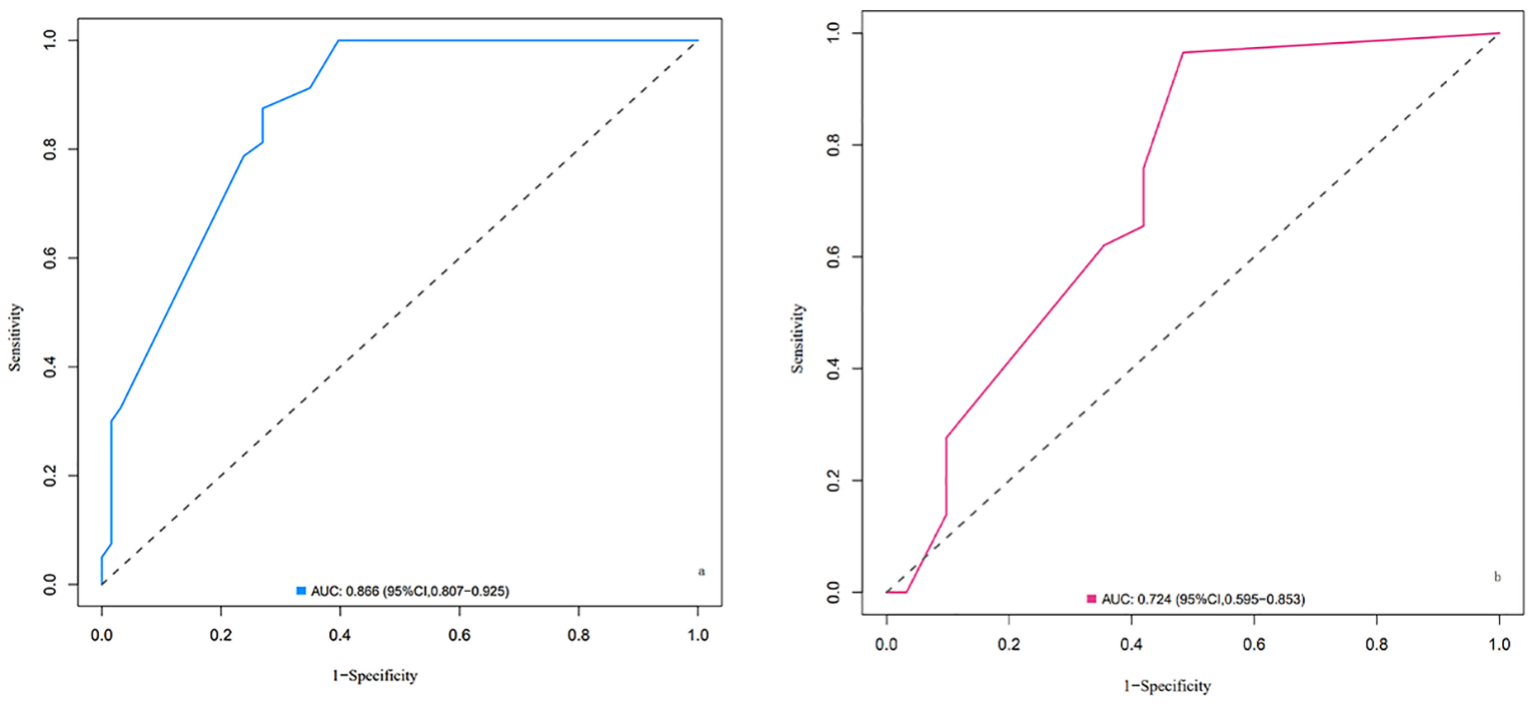
ROC curves of the nomogram for LARS risk prediction in the training cohort **(A)** and validation cohort **(B)**. **(A)** ROC curve for the training cohort. The area under the curve (AUC) is 0.866 (95% CI: 0.807–0.925), indicating excellent discriminative ability of the model in distinguishing between patients with and without LARS. **(B)** ROC curve for the validation cohort. The AUC is 0.724 (95% CI: 0.595–0.853), demonstrating good predictive performance of the model in an independent dataset. Explanation: The x-axis represents 1-specificity (false positive rate), and the y-axis represents sensitivity (true positive rate). The diagonal dashed line represents a random classifier with no discriminatory ability (AUC = 0.5). The farther the curve is above this line, the better the model’s predictive performance.

ROC curves were plotted for the two groups of patients. The AUC of the column-line chart predictive model in the experimental group was 0.866 (95% CI: 0.807~0.925), while in the validation group, it was 0.724 (95% CI: 0.595~0.853). The C-index of the predictive model in the two patient groups was 0.873 and 0.723, respectively, indicating that the model has good discriminative ability, as shown in **Figure 2**.

### 3.6 Calibration curves of LARS occurrence in training and validation cohorts

**Figure 3:**
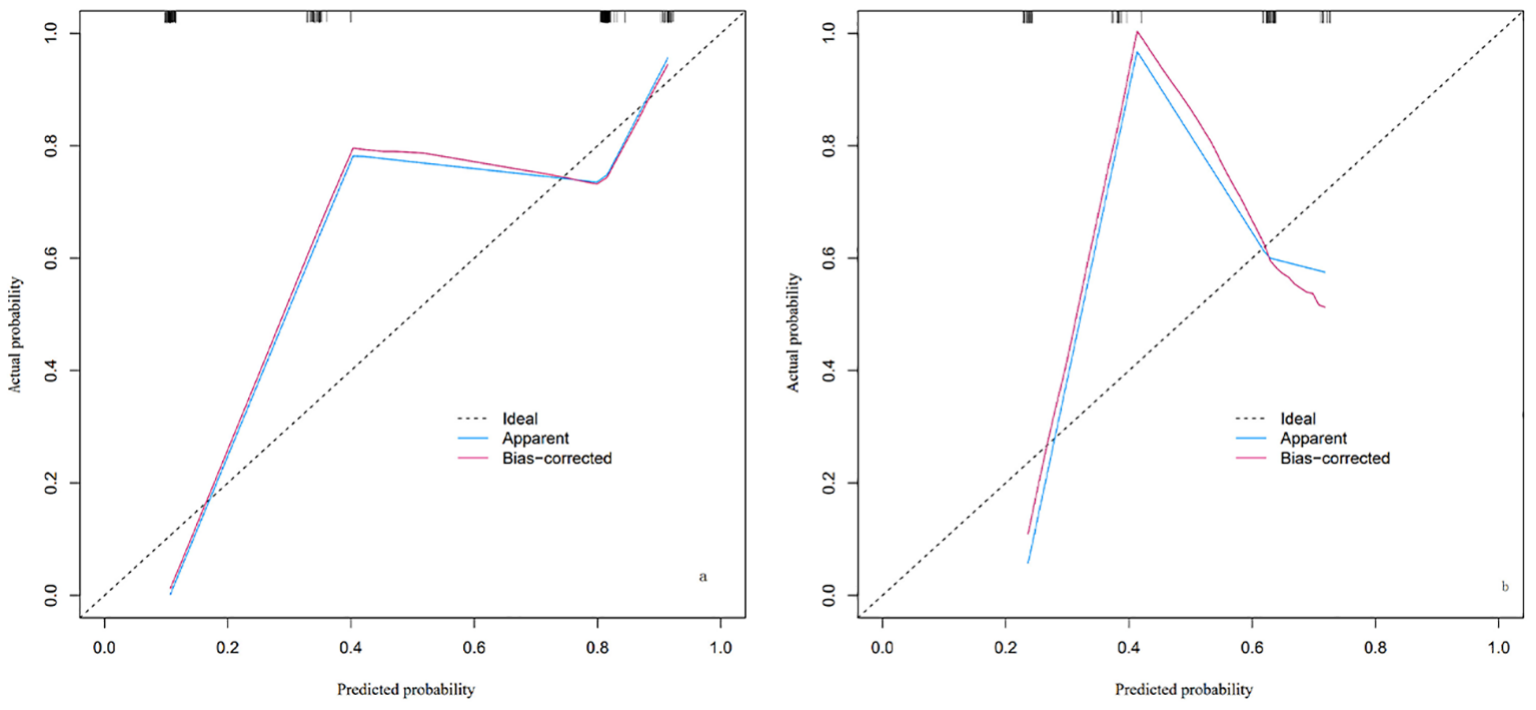
Calibration plot of the nomogram for LARS risk in the training cohort **(A)** and validation cohort **(B)**. The plot includes three lines: the Ideal line (dashed), representing perfect agreement between predicted and actual probabilities; the Apparent line (blue), showing the model’s calibration based on the training cohort; and the Bias-corrected line (red), adjusted through cross-validation to account for potential overfitting. The closer the Apparent and Bias-corrected lines are to the Ideal line, the better the model’s calibration. By observing where specific predicted probabilities intersect with the Apparent or Bias-corrected lines, the model’s predictive accuracy at different probability levels can be assessed.

The calibration of the predictive model in the training and validation cohorts was evaluated using the Hosmer-Lemeshow goodness-of-fit test. This test compares the predicted probabilities with the actual outcomes across different groups, with a P-value >0.05 indicating a good fit. In our study, the P-values for both the training and validation cohorts were >0.05, suggesting good calibration of the model, as shown in **Figure 3**.

### 3.7 Clinical value of the nomogram prediction model

**Figure 4:**
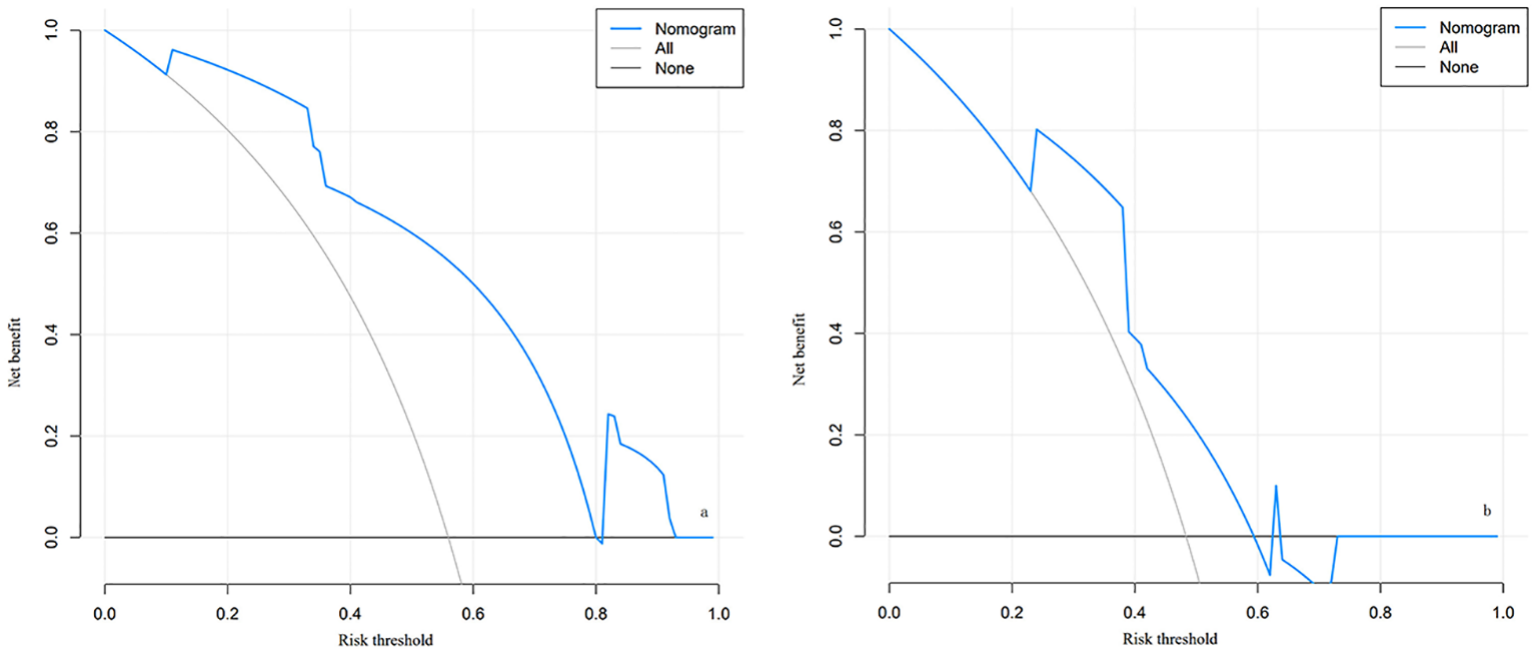
The decision curve analysis (DCA) for the nomogram of LARS in the training cohort **(A)** and validation cohort **(B)**. **(A)** Decision curve for the training cohort; **(B)** Decision curve for the validation cohort. The curves illustrate the net benefit across different risk thresholds. The blue curve represents the net benefit derived from the nomogram model, the “All” curve (gray) assumes all patients develop LARS, and the “None” curve (black) assumes no patients develop LARS. Explanation: When the risk threshold is between 0.11 and 0.80, the nomogram model (blue curve) demonstrates a higher net benefit, indicating good clinical applicability in both the training and validation cohorts. A higher net benefit suggests greater utility of the model in guiding patient management and intervention.

The decision curve analysis (DCA) curve demonstrates that there is a high clinical net benefit when the threshold probability ranges from 0.11 to 0.80. Net benefit values are clinically significant when they are greater than 0, and the smaller the threshold probability, the greater the net benefit, as shown in **Figure 4**.

## 4 Discussion

Rectal cancer is one of the most common malignancies in the digestive tract, with its incidence and mortality rates increasing annually worldwide (7). For rectal surgeons, achieving complete resection of rectal tumors while preserving anal function in patients is a focal point of concern (8). In recent years, with ongoing research into the biological characteristics of rectal cancer and advancements in surgical techniques, particularly the progress in laparoscopic techniques and adjuvant therapies such as radiotherapy and chemotherapy, the treatment of rectal cancer has not only improved resection rates and reduced postoperative recurrence rates but also enhanced patients’ quality of life (9). However, studies focusing on mixed-gender cohorts may overlook the specific challenges faced by male rectal cancer patients. Factors such as a narrower pelvic cavity and more severe rectal fibrosis induced by preoperative radiotherapy make anal preservation and functional recovery more difficult in this subgroup (10, 11). Approximately 25% to 90% of patients experience varying degrees of anal dysfunction after rectal cancer surgery, known as LARS (12, 13). LARS encompasses symptoms such as fecal incontinence, urgency, and frequency, with a prolonged recovery period, and in some cases, symptoms persist, severely affecting patients’ postoperative quality of life (14). Therefore, enhancing understanding of the risk factors for LARS development and recovery status after rectal cancer surgery has become an urgent requirement in the comprehensive management of rectal cancer. However, current treatment measures for LARS remain insufficient, with clinical management predominantly relying on empirical approaches. Actively identifying potential risk factors for LARS and implementing corresponding preventive interventions can help reduce its occurrence, thus alleviating its impact on patients’ postoperative quality of life.

This study evaluated 203 male patients with mid-low rectal cancer who underwent neoadjuvant therapy combined with laparoscopic TME using the LARS scale assessment. The incidence of LARS was found to be 53.6%, consistent with the range reported in similar studies (35–37). LARS-related symptoms gradually eased with postoperative time extension but were difficult to completely eliminate. The results of this study indicate that a BMI ≥ 25 kg/m^2^, tumor distance from the anal verge <5 cm, radiotherapy, and anastomotic leakage are high-risk factors for the occurrence of LARS in male patients with mid-low rectal cancer undergoing neoadjuvant therapy combined with laparoscopic TME. (1) BMI ≥ 25 kg/m²: Overweight or obesity increases the risk of perioperative complications in rectal diseases, such as wound infection and anastomotic leakage. This is particularly significant in surgeries for mid-low rectal cancer, where increased visceral fat volume further narrows the already narrow pelvic space in males (15). This can lead to blurred surgical vision, increased blood loss, and prolonged surgery duration. These factors add to the challenges of rectal cancer surgery and impact overall postoperative outcomes, including bowel function (16). Although patients with BMI ≥ 25 kg/m^2^ have a higher risk of developing LARS postoperatively in this study, the sample size was limited, necessitating further investigation. (2) Tumor distance from the anal verge <5cm: In order to ensure an adequate margin, the anastomosis in surgeries for low rectal cancer is typically positioned below the level of the anorectal ring. However, this may potentially damage the anal sphincter and its surrounding nerves. Damage to the internal anal sphincter can result in passive fecal incontinence, where rectal contents leak out of the anus without sensation. Damage to the external anal sphincter typically causes urgency fecal incontinence, where the leakage is perceived but the patient cannot control it. Additionally, if the descending neural pathway is impaired, it leads to the loss of rectoanal reflex, disrupting the integrity of rectal coordinated movements. Consequently, when the rectum expands, there is an inability to spontaneously relax or contract the sphincter based on environmental conditions, thus controlling defecation (17). (3) Radiotherapy: Relevant studies indicate that preoperative radiotherapy helps improve sphincter preservation rates, reduce local tumor recurrence rates, and decrease distant metastasis rates in rectal cancer patients. However, radiotherapy is an invasive procedure that may increase the occurrence of postoperative complications (18). Preoperative radiotherapy may lead to fibrosis of normal tissues surrounding the lesion, thereby reducing the compliance of the rectal anal canal. This could potentially cause damage to the pelvic autonomic nerves and rectal nerves, resulting in the disappearance of rectoanal inhibitory reflex, decreased anal resting pressure, and ultimately triggering the occurrence of LARS (19, 20). (4) Anastomotic leakage: This study identified anastomotic leakage as one of the independent risk factors for the occurrence of LARS after sphincter-preserving surgery for rectal cancer. Anastomotic leakage is a severe complication following surgery for rectal cancer, where its formation may lead to the development of local abscesses, subsequent inflammatory responses, fibroproliferation of the rectal wall, ultimately resulting in anastomotic stricture and decreased rectal wall compliance, thereby increasing postoperative bowel frequency. Upon subsequent healing, anastomotic leakage may result in significant scar formation, impacting the volume and motility of the neo-rectum. Even after conservative treatment, it may lead to a decrease in rectal reservoir, consequently causing abnormal gas and stool evacuation, thus further increasing the risk of developing LARS (21, 22).

Providing personalized intervention strategies for patients at high risk of LARS is crucial. For patients with a higher BMI, preoperative nutrition and weight management may help reduce postoperative complications (23–25). For patients with tumors located closer to the anal verge, surgical techniques should aim to preserve the integrity of the anal sphincter as much as possible, and postoperative functional recovery plans should be considered (26–28). For patients receiving radiotherapy, special attention should be given to complications potentially caused by radiation, and symptoms should be alleviated postoperatively through medication or rehabilitation interventions (29–31). Additionally, for patients with anastomotic leaks, early diagnosis and proactive interventions can help reduce the long-term impact on postoperative bowel function (32–34). For the treatment of LARS in post-rectal cancer surgery patients, various interventions can be employed to improve anorectal function and quality of life. The primary treatment methods include pelvic floor rehabilitation therapies such as pelvic floor muscle training, biofeedback therapy, and rectal balloon training. These approaches can significantly enhance the strength of the pelvic floor and perianal muscles, thereby alleviating symptoms such as fecal incontinence and increased bowel frequency (26, 27, 38–40). For patients with more severe symptoms, neuromodulation techniques such as sacral nerve stimulation and transcutaneous tibial nerve stimulation can be utilized to further improve bowel control (41, 42). Additionally, dietary interventions and pharmacotherapy can effectively manage bowel symptoms, thereby enhancing patients’ quality of life (19, 27, 43). Additionally, it is important to note that patients undergoing TME after chemoradiotherapy often experience other complications, including urinary and sexual dysfunction, fertility limitations, psychological issues, and a general impairment of quality of life (44, 45). To address these multifaceted challenges, a systematic multidisciplinary approach should be adopted as a standard protocol in the management of patients scheduled for low anterior resection. This approach involves collaboration between colorectal surgeons, urologists, gynecologists, psychologists, and rehabilitation specialists to comprehensively manage both the physical and psychological aspects of recovery. By offering integrated care, we can improve the overall well-being of patients, ensuring that they receive optimal support throughout their recovery process (31, 46).

Limitations: Firstly, due to the retrospective design of this study, there is a possibility of information bias and selection bias. Secondly, the sample of this study is derived from a single medical institution, which may limit the generalizability of the results due to regional and ethnic differences. Additionally, this study only considered some factors that may affect LARS, while other potential factors were not included in the analysis. Importantly, the predictive model developed is primarily tailored for male patients, and its applicability to females requires further investigation. Lastly, this study did not further evaluate the effectiveness of therapeutic interventions. Further research is needed to validate how to effectively prevent and manage LARS. Although the LARS questionnaire is widely used in clinical practice, its specificity is limited and may underestimate the impact of postoperative quality of life and bowel dysfunction. To more comprehensively assess these factors, future research and clinical practice may need to incorporate other more sensitive and specific assessment tools to optimize the evaluation and management of postoperative symptoms in patients.

## 5 Conclusion

In summary, BMI ≥25 kg/m², tumor distance from the anal verge <5cm, radiation therapy, and anastomotic leakage are independent risk factors for the occurrence of LARS in male patients with mid-low rectal cancer undergoing neoadjuvant therapy combined with laparoscopic TME. Recognizing these factors preoperatively is critical for personalized patient counseling and the implementation of targeted preventive measures, thereby potentially reducing the incidence and severity of LARS in clinical practice.

## Data Availability

The raw data supporting the conclusions of this article will be made available by the authors, without undue reservation.
